# Heterogeneous mosquito exposure increases *Plasmodium vivax* and *Plasmodium falciparum* co-infections: a modelling study

**DOI:** 10.1098/rspb.2024.2061

**Published:** 2024-12-04

**Authors:** Mathilde Grimée, Aimee R. Taylor, Michael T. White

**Affiliations:** ^1^ Infectious Disease Epidemiology and Analytics G5 Unit, Institut Pasteur, Université Paris Cité, Paris, France; ^2^ Sorbonne Université, Collège doctoral, Paris, France

**Keywords:** modelling, *Plasmodium vivax* and *Plasmodium falciparum*, co-infection, heterogeneous, mosquitoes, exposure

## Abstract

In malaria-endemic regions, *Plasmodium vivax* and *Plasmodium falciparum* coexist and may interact. For instance, fevers induced by *P. falciparum* might activate dormant *P. vivax* parasites and concurrent radical cure of both species has been proposed to prevent relapses. Heterogeneous mosquito exposure may contribute to the dependence of both parasites. We conducted a literature review on their respective prevalence and that of co-infections. The data revealed a positive correlation between *P. vivax* and *P. falciparum* prevalence, and co-infection prevalences exceeding expectations assuming infections occur independently. We used the review data to fit a compartmental model of co-infections that features heterogenous mosquito exposure. The fit suggests that heterogeneous exposure sufficiently explains the observed departure from independence. Finally, we performed simulations under the model assessing the impact on *P. vivax* prevalence of the activation-by-fever hypothesis and the radical cure proposition. We demonstrated a moderate impact of allowing *P. falciparum* fevers to reactivate *P. vivax* and a substantial impact of treating *P. falciparum* cases with radical cure. Our model highlights dependence between *P. falciparum* and *P. vivax* and emphasizes the influence of heterogeneous mosquito exposure. This simple framework can inform the design of more complex models assessing integrated malaria control strategies in coendemic regions.

## 1. Introduction

Malaria, a mosquito-borne disease caused by *Plasmodium* parasites, remains a significant global health challenge. *Plasmodium falciparum* and *Plasmodium vivax* are the two most prevalent and clinically significant species, co-circulating in regions of Southeast Asia, East Africa, South America and Central America [1]. While *P. falciparum* is often associated with more severe manifestations of the disease, including cerebral malaria and severe anaemia, *P. vivax* presents unique challenges due to its ability to form dormant liver stages, known as hypnozoites, causing relapses weeks to months after a primary infection [2]. Outside of Africa, as endemicity declines, an increasing proportion of malaria cases are caused by *P. vivax* as opposed to the historically predominant *P. falciparum* [1,3]*.* Co-infections with both species are common, despite underreporting [4]. Studying the interactions between *P. vivax* and *P. falciparum* through laboratory and field studies has a long-standing history. A common metric on which those studies rely to establish interaction is the cross sectionally observed excess or deficit in co-infections relative to what is expected under the null hypothesis that *P. vivax* and *P. falciparum* infections are acquired independently. Using this metric, evidence supporting both an antagonistic relationship, with an observed deficit in co-infections, and supporting a synergistic relationship between the parasites, with an observed excess in co-infections, can be found in the literature, as summarized in a 1988 review [5]. To explain the observed deficit in co-infections, it was proposed that host cross-immunity could mediate mutual suppression. However, it has also been proposed early on that a deficit in co-infections could be explained simply by their artefactual underreporting in studies using diagnosis by microscopy [6]. In terms of the observed excess in co-infections, it was proposed that rather than being caused by a true interaction between *P. vivax* and *P. falciparum*, it could be explained by risk heterogeneity, which would concentrate both species onto certain susceptible hosts via genetic or behavioural factors [7]. Furthermore, Hamelin *et al*. [8] and Domenech de Cellès *et al*. [9] call for caution when drawing conclusions on pathogen interactions based on the relative prevalence of co-infection, showing that non-interacting or even negatively interacting pathogens could, under the right circumstances, lead to an excess in co-infection. All in all, understanding the co-infection dynamics of *P. falciparum* and *P. vivax* will contribute to the design of more effective integrated control and elimination strategies.

Mathematical models have provided valuable insights into the transmission dynamics of vector-borne diseases [10]. Mathematical models of *P. falciparum* transmission rely on the theoretical foundation of the Ross–Macdonald model describing the transmission of a pathogen between human and vector hosts [11–17]. Classic compartmental models and agent-based simulation models have been widely employed to simulate the spread of *P. falciparum* within populations, providing valuable insights into the impact of interventions such as vector control and antimalarial treatment. Most notable are the models developed at Imperial College [18,19], SwissTPH [20] and Institute for Disease Modelling [21]. Mathematical models of malaria transmission have been adapted to incorporate the dynamics of *P. vivax* hypnozoite activation, subsequent relapse and thus transmission [17,22–25]. A review article [26] of malaria transmission models published between 1998 and 2018 found only two models tracking both *P. falciparum* and *P. vivax* infections [27,28] with several other models of co-infection identified outside of the review [29–32].

An important limitation of these models is that they do not account for heterogeneous mosquito biting exposure, a key driver of co-infection dynamics [33–36]. Individuals experiencing higher biting rates through factors, such as human behaviour, host attractiveness and environmental conditions, are more exposed to malaria infection [37], possibly leading to localized clusters of transmission. This differential exposure to infected mosquitos affects population-level transmission dynamics [35,37,38] and creates an increased risk of co-infection with multiple *Plasmodium* species for some individuals [5].

While existing mathematical models have provided valuable insights into malaria transmission dynamics, there is a clear need for models that feature both the complexities of co-infections and heterogeneity in biting exposure. In this study, we developed a simple compartmental susceptible–infected–recovered-type model of *P. falciparum* and *P. vivax* with heterogeneity in exposure to infectious mosquito bites. We fitted the model to literature review data using approximate Bayesian computation (ABC). Through simulations and scenario analyses, we demonstrated how co-infection patterns can be explained by heterogeneous mosquito exposure. We investigated the epidemiological consequences of a biological interaction between these species, whereby fever caused by *P. falciparum* infection may trigger a *P. vivax* relapse. Finally, we explored how these species respond to integrated treatment regimens.

The novelty of this contribution lies in its parsimony: including heterogeneous mosquito exposure into a simple compartmental model is sufficient to qualitatively capture co-infection dynamics. This simple approach can inform the design of more complex models quantitatively assessing integrated malaria control strategies in coendemic regions.

## 2. Material and methods

### (a) Data review

We performed a literature review on PubMed to collect data from field epidemiology studies recording *P. vivax*, *P. falciparum* and co-infection prevalence, among the general population. Specifically, we defined those studies as cross-sectional and active infection-detection studies performed in communities, unrestricted by age, sex or symptomatic status. The keywords we used were

Title: (Malaria OR Vivax OR Falciparum) NOT (Health-care facility OR Hospital OR Health centre OR Systematic Review OR Meta*)

Abstract: Prevalence OR Distribution OR Epidemiology OR Cross-section

Text: Vivax AND Falciparum

After title and abstract screening with Rayyan [39], we screened the main text and extracted the *P. vivax*, *P. falciparum* and co-infection prevalence from each study, as well as the sample size and diagnostic method used to generate prevalence. We excluded studies that failed to report all those metrics or that did not screen the general population. A spreadsheet of the details of all the studies retained after title and abstract screening is available as a supplementary file. We first evaluated the correlation between *P. vivax* and *P. falciparum* prevalence using a Pearson correlation test weighted by study size and evaluated at a significance level α=0.05. Then, we defined different metrics related to the coupled dynamics of *P. vivax*, *P. falciparum* and co-infections. First, we introduced the notion of expected co-infection prevalence under independence of *P. vivax* and *P. falciparum*. That is, we assumed that if *P. vivax* and *P. falciparum* transmission are independent, co-infection prevalence should be the product of *P. falciparum* and *P. vivax* prevalence. We then defined the proportional excess co-infection prevalence e as the difference between observed and expected co-infection prevalence, $p_{FV}^{O}$and $p_{{FV}^{E}}$, respectively, normalized by different measures of local transmission intensity: *P. vivax* prevalence (i), *P. falciparum* prevalence (ii) or overall malaria prevalence (iii):


(2.1) $$e_{V}=\frac{p_{FV}^{O}-\;p_{FV}^{E}}{p_{V}},$$

(2.2) $$e_{F}=\;\frac{p_{FV}^{O}-\;p_{FV}^{E}}{p_{F}},$$

(2.3) $$e_{FV}=\;\frac{p_{FV}^{O}-\;p_{FV}^{E}}{p_{F}^{O}+\;p_{V}^{O}-\;p_{FV}^{O}},$$

where $p_{V}^{O}\geq p_{FV}^{O}$ and $p_{F}^{O}\geq p_{FV}^{O}$ are the observed *P. vivax* and *P. falciparum* prevalence, respectively.

### (b) Transmission model

To capture the dynamics of *P. vivax* and *P. falciparum* in humans and in mosquitos, we used two coupled deterministic systems of ordinary differential equations: one for humans and one for mosquitos. For the human system, we considered three binary states of infection: infection with *P. falciparum*, blood-stage infection with *P. vivax* and liver-stage infection with *P. vivax* hypnozoites. All combinations of these states are possible, leading to eight compartments in the model. Infection by a mosquito infected with *P. vivax* causes humans to move to an infected compartment for *P. vivax* blood and liver stages at rate $\lambda_{h_{V}}$. Infection by a mosquito infected with *P. falciparum* causes humans to move into an infected compartment for *P. falciparum* at rate $\lambda_{h_{F}}$. Infection by a mosquito infected with both species causes humans to move into the compartment positive for all three infection states at rate $\lambda_{h_{FV}}$. Humans with blood-stage infections recover at rate $r_{F}$ or $r_{V}$, respectively. Hypnozoites activate at rate f and die at rate γ.

The mosquito system follows a susceptible–exposed–infected dynamic. We considered all combinations of three infection states (S, E and I) for the two species, leading to nine compartments in the model. Infection by feeding on a human infected with blood-stage *P. vivax* causes mosquitos to move into an exposed compartment for *P. vivax* at rate $\lambda_{m_{V}}$. Infection by feeding on a human infected with *P. falciparum* causes mosquitos to move into an exposed compartment for *P. falciparum* at rate $\lambda_{m_{F}}$. Infection by feeding on a human infected with blood-stage parasites of both species causes mosquitos to move in the exposed compartment for both at rate $\lambda_{m_{FV}}$. Mosquitos move from exposed to infected at rate $\eta_{V}$ or $\eta_{F}$. Two compartments account for being simultaneously infected with *P. vivax* and exposed to *P. falciparum* or vice-versa. Mosquitos are born and die at rate μ.

Equations for the systems and graphical representation of the compartments and dynamics are shown in the electronic supplementary material, figures S1 and S2.

### (c) Heterogeneity

We stratified the human compartments into nine groups accounting for different levels of exposure to infectious mosquito bites. We assumed that the number of bites per day, X, are log-normally distributed with mean 1 and variance $\sigma^{2}$: $X\;∼\;logN\;\left(1,\;\sigma^{2}\right)$. Log-bites, $Z=\log\left(X\right)$, are thus normally distributed, $Z∼N\left(1,\sigma^{2}\right)$ (electronic supplementary material, figure S3). The larger the variance, the more bites cluster in on smaller parts of the population (electronic supplementary material, figure S4). We discretize the normal distribution using the Gauss–Hermite quadrature approach, described elsewhere [23]. This procedure outputs weights ($w_{i}$) used to set the size of the nine strata of the compartmental model, and abscissae ($x_{i}=\exp\left(z_{i}\right)$) to scale the force of infection. The subscript i∈1,2,…9 is a population stratum index. The force of infection for *P. vivax*, *P. falciparum* and co-inoculation on humans was adjusted with the scaling factors $x_{i}$ as follows:


$$\lambda_{h_{F_{i}}}=x_{i}\cdot a\cdot m\cdot b_{F}\left(I_{m_{F}}+I_{m_{F}}E_{m_{V}}\right)+b_{F}\left(1-b_{V}\right)I_{m_{FV}},$$

$$\lambda_{h_{V_{i}}}=x_{i}\cdot a\;\cdot m\cdot b_{V}\left(I_{m_{V}}+I_{m_{V}}E_{m_{F}}\right)+b_{V}\left(1-b_{F}\right)I_{m_{FV}},$$

$$\lambda_{h_{{FV}_{i}}}=x_{i}\cdot a\cdot m\cdot b_{F}b_{V}I_{m_{FV}}.$$

The force of infection on mosquitos was adjusted as follows:


$$\lambda_{m_{F}}=a\cdot \varphi \cdot c_{F}\cdot \sum_{i}x_{i}w_{i}\left(I_{{h_{F}}_{i}}+I_{{h_{FH}}_{i}}\right)+\varphi \cdot c_{F}\left(1-\left(2-\varphi \right)\cdot c_{V}\right)\cdot \sum_{i}x_{i}w_{i}\left(I_{{h_{FV}}_{i}}+I_{{h_{FVH}}_{i}}\right),$$

$$\lambda_{m_{V}}=a\cdot \left(2-\varphi \right)\cdot c_{V}\cdot \sum_{i}x_{i}w_{i}\left(I_{{h_{V}}_{i}}+I_{{h_{VH}}_{i}}\right)+\left(2-\varphi \right)\cdot c_{V}\left(1-\varphi \;\cdot c_{F}\right)\cdot \sum_{i}x_{i}w_{i}\left(I_{{h_{FV}}_{i}}+I_{{h_{FVH}}_{i}}\right),$$

$$\lambda_{m_{FV}}=a\cdot \varphi \cdot c_{F}\cdot \left(2-\varphi \right)\cdot c_{V}\cdot \sum_{i}z_{i}x_{i}\left(I_{{h_{FV}}_{i}}+I_{{h_{FVH}}_{i}}\right).$$

Symbol and parameter descriptions for the six equations above can be found in electronic supplementary material, table S1.

### (d) Model fitting

We fit the model to the polymerase chain reaction (PCR) data points identified in the literature review to generate parameter estimates for $\sigma^{2}$, m and φ using ABC. The parameter $\sigma^{2}$ is the variance of the heterogeneity distribution, m is the mosquito density and φ is the relative vector competence (i.e. the relative ability of a mosquito to transmit one species versus the other). All other parameters had fixed values (electronic supplementary material, table S1), and we tested the dependence of our conclusions on those values with a sensitivity analysis (electronic supplementary material, figure S5). We sampled 100 000 draws from the following prior distributions:


$$\sigma^{2}∼Exp\;\left(1,\;1\right),$$

m∼Exp(1, 1),

φ∼Unif(0, 2).

We plugged each of the 100 000 independent draws into the model and simulated *P. vivax*, *P. falciparum* and co-infection prevalence by running the model to equilibrium, which is shown in electronic supplementary material, figure S6. For each of the i PCR-data points in parallel, we added measurement error to each of the j=1,…, 100000 prevalence trios by drawing a simulated data point drawn from $Multinom(n_{i},p_{j})$ where $n_{i}$ is the sample size used to generate the PCR data point and $p_{j}$ is the simulated prevalence trio. Then, we computed the Euclidian distance between the PCR data point and each simulated data point on the logit scale and selected its 100 nearest neighbours. We finally assessed how well simulated data points from the fitted heterogeneous model recreate the distribution of the true set of PCR data points, compared with a model without heterogeneity ($\sigma^{2}=0$), by plotting an illustrative set of simulated data points from the ensemble of nearest neighbours. The parameter values that generated the i sets of 100 nearest neighbours were combined into a single set of parameter trios, which can be seen as a numerical approximation of the ensemble of posteriors. Parameter estimates used for the following simulations were based on the marginal 25%, 50% and 75% quantiles of the numerical approximation of the posterior ensemble.

### (e) Scenario simulations

We performed scenario simulations for three possible epidemiological settings: low transmission with high heterogeneity (25% and 75% quantiles of m and $\sigma^{2}$, respectively), medium transmission with medium heterogeneity (50% quantiles of both $m\;and\;\sigma^{2}$) and high transmission with low heterogeneity (75% and 25% quantiles of m and $\sigma^{2}$, respectively). We chose a single value for φ, the median of the numerical approximation of the posterior ensemble.

### (f) Hypnozoite activation-by-fever

We explored the hypothesis that *P. falciparum*-induced fevers could activate *P. vivax* hypnozoites. In addition to the spontaneous *P. vivax* relapse rate f originating from hypnozoite-infected compartments, we introduced an additional relapse rate triggered by *P. falciparum* fevers. A proportion $\pi_{F}\pi_{T}$ of the force of infection $\lambda_{F}$ exiting from hypnozoite-infected compartments is deviated towards the compartment infected with hypnozoites, and blood-stage *P. falciparum* and blood-stage *P. vivax*, while the remainder $1-\left(\pi_{F}\pi_{T}\right)$ continues as usual towards the compartment infected with hypnozoites and *P. falciparum* only. These dynamics are illustrated in the electronic supplementary material, figure S7. The parameter $\pi_{F}$ represents the proportion of *P. falciparum* infections that are febrile, and $\pi_{T}$ represents the hypothetical proportion of *P. falciparum* infections that would trigger a *P. vivax* relapse. Given the unknown nature of the parameter $\pi_{T}$, we performed a sensitivity analysis by setting the parameter to 0.1, 0.5 or 1. As seen in electronic supplementary material, table S1, we set $\pi_{F}=0.5$.

### (g) Treatment

We implemented treatment through a partial instantaneous blocking of a given force of infection. A proportion πθ of the force of infection is deviated towards a susceptible compartment, and the residual proportion 1-πθ continues towards an infected compartment as usual. The parameter π is the proportion of infections that are febrile, and the parameter θ is a composite parameter, encompassing the various factors that govern successful treatment (health care access, adherence, treatment effectiveness, etc.). We differentiated between $\pi_{F}$ and $\pi_{V}$ as the febrile proportions for *P. falciparum* and *P. vivax,* respectively. To simulate the standard treatment guidelines, we considered that a new *P. vivax* infection would be treated with radical cure, clearing hypnozoites and blood-stage parasites, and a new *P. falciparum* infection would be treated with blood-stage-only treatment. When a new infection is treated, any concurrent infection would also be cured due to the treatment. For a new co-inoculation, a proportion $\pi_{V}\theta$ is blocked with radical cure, and $\pi_{F}\;\left(1-\pi_{V}\right)\theta$ with only blood-stage-only treatment. The treatment dynamics are illustrated in the electronic supplementary material, figure S8. As seen in electronic supplementary material, table S1, we set $\pi_{F}=0.5$, $\pi_{V\;}=0.1$ and θ=0.25.

## 3. Results

### (a) Data review

We collected data from field epidemiology studies recording population prevalence of *P. vivax*, *P. falciparum* and co-infection. Our search terms identified 759 articles on PubMed. After abstract screening, we obtained 125 articles, among which 37 reported three-way prevalence. From the 37 articles, we extracted 104 data points (each a prevalence trio). The 104 data points included 58 data points that were generated using PCR, which is more precise than other methods such as microscopy or rapid diagnostic tests. When establishing the presence of a species by microscopy, one is likely to stop the test once a first species is observed thereby discarding the potential presence of a second or third species [6]. This bias would likely lead to a strong underestimation of co-infection prevalence. We therefore present these data in the electronic supplementary material, figure S9 but exclude them from the main analyses.

**Figure 1.**
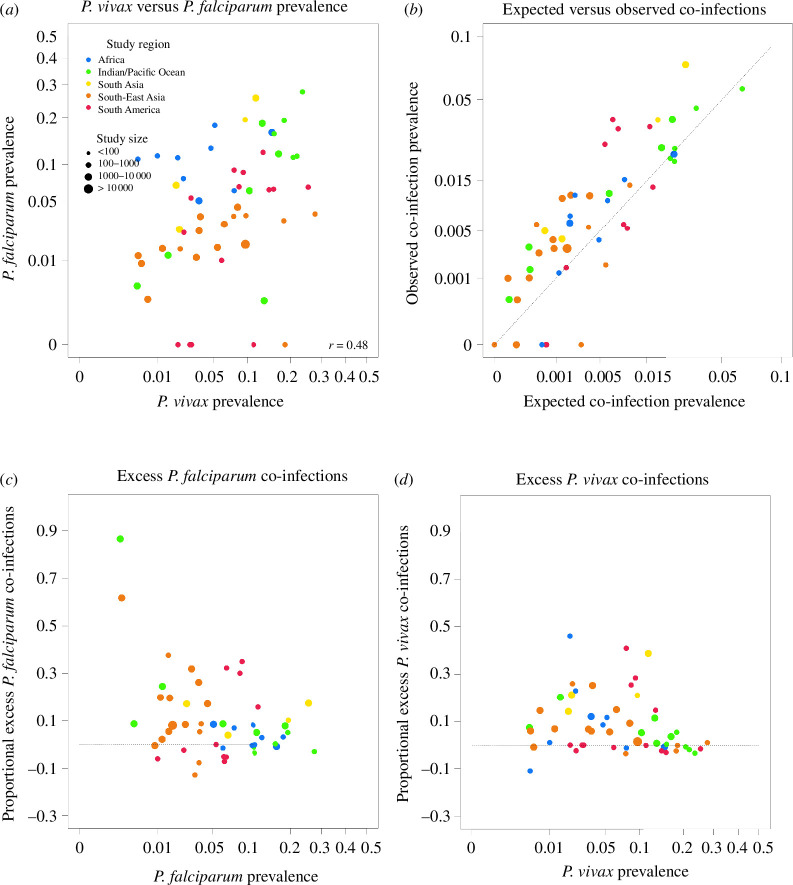
Literature review of *P. falciparum* and *P. vivax* prevalence by PCR. Each data point is obtained by PCR. (*a*) Relationship between *P. vivax* prevalence and *P. falciparum* prevalence. The annotation r=0.48, is the Pearson correlation estimate, weighted by study size. (*b*) Expected versus observed co-infection prevalence. Expected co-infection prevalence is the product of *P. falciparum* prevalence and *P. vivax* prevalence. The dashed diagonal line represents equality between expected and observed co-infection prevalence. Most data points lie above the line, indicating an excess in co-infections. (*c*) Excess co-infection prevalence, proportional to *P. falciparum* prevalence and against *P. falciparum* prevalence. The dashed line corresponds to zero excess co-infections. (*d*) Excess co-infection prevalence, proportional to *P. vivax* prevalence and against *P. vivax* prevalence. The dashed line corresponds to zero excess co-infections.

There is a positive linear relationship between *P. vivax* and *P. falciparum* PCR-prevalence (figure 1*a*). Figure 1*b* illustrates the difference between co-infection prevalence expected under independence, defined as the product of *P. vivax* and *P. falciparum* prevalence, and observed co-infection prevalence. Most of the data points exist above or at the line of equality, signifying more co-infections than expected under independence. To explore whether that excess is related to transmission intensity, we computed the proportional excess in co-infection prevalence and plotted it against species-specific prevalence (figure 1*c*,1*d*). Departure from independence varies more when prevalence is lower.

### (b) Model fitting and validation

We estimated the values of three model parameters $\sigma^{2}$, m and φ, by fitting our model of co-infection with heterogeneity to the 58 PCR data points. The marginal 25%, 50% and 75% quantiles, respectively, of the parameters used to generate the combined 5 800 nearest-neighbour simulated points are 0.67, 1.53 and 2.4 ($\sigma^{2})$; 0.27, 0.34 and 0.63 (m); and 0.85, 1.01 and 1.15 (φ). While for most data points, the pairwise marginal posteriors are well behaved (electronic supplementary material, figure S11a,b), for some points where any of the three prevalence values approaches 0, a range of parameter pairs generate the same prevalence triplet, indicating issues of identifiability (electronic supplementary material, figure S11c).

**Figure 2.**
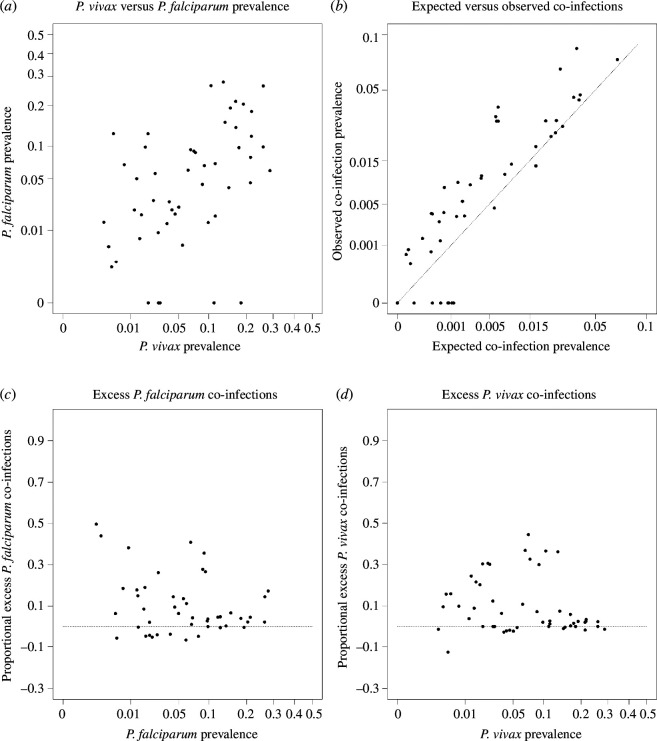
Model validation. Simulated dataset generated by drawing an illustrative set of 58 simulated data points uniformly at random from the full set of 5 800 simulated data points. The latter can be found in the electronic supplementary material, figure S10. Each panel is generated in the same way as figure 1.

We evaluated our model’s capacity to generate simulated data that resemble real data by plotting 58 simulated points drawn uniformly at random from the 5 800 nearest neighbours (figure 2). This illustrative set of 58 simulated data points resembles the distribution of the true set of 58 PCR data points, indicating that our simple model of co-infection with heterogeneity captures departure from independence in observed co-infection prevalence. Draws from the model without heterogeneity ($\sigma^{2}\;=\;0$) show that some excess co-infections are still expected but not to the extent observed in the data (electronic supplementary material, figure S12).

### (c) Scenario simulations

We used the aforementioned parameter estimates to explore the activation-by-fever hypothesis and the radical cure proposition under three epidemiologically plausible conditions. The simulations for all other combinations of the quartiles of m and $\sigma^{2}$ are shown in the electronic supplementary material, figures S13, S14.

**Figure 3.**
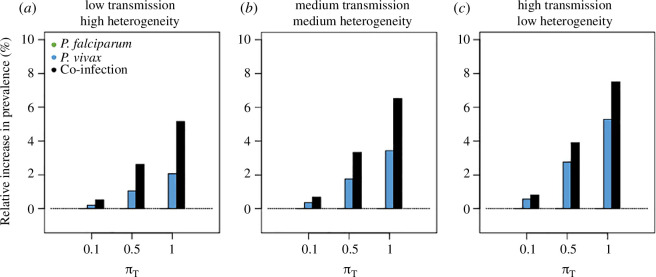
Febrile triggering of *P. vivax* relapse. Relative increase in *P. falciparum*, *P. vivax* and co-infection prevalence caused by a scenario where *P. falciparum*-induced fevers activate hypnozoites, compared with a scenario without. The highest increase is seen in co-infection (+5.3%) and *P. vivax* prevalence (+7.5%) in the panel with medium transmission and heterogeneity with a parameter $\pi_{T}=1$. (*a*) This panel shows low transmission and high heterogeneity with m=0.27 and $\sigma^{2}=2.4$. (*b*) This panel shows medium transmission and medium heterogeneity with m=0.34 and $\sigma^{2}=1.53$. (*c*) This panel shows high transmission and low heterogeneity with m=0.63 and $\sigma^{2}=0.6$.

In figure 3, we explore the epidemiological consequences of triggering of *P. vivax* relapses by febrile *P. falciparum* infections in the absence of treatment. Allowing *P. falciparum*-induced fever to trigger relapses has the greatest effect in the setting with high transmission and low heterogeneity (figure 3*c*). In this setting, allowing all *P. falciparum*-induced fevers to activate hypnozoites ($\pi_{T}=1$), the scenario most favourable to the hypothesis, would increase *P. vivax* prevalence from 39.9% to 42.0%, that is by at most 5.3% and co-infection prevalence from 20.7% to 22.3%, that is by 7.5%. While this model does not provide evidence on the likelihood of febrile *P. falciparum* triggering *P. vivax* relapses, we can conclude that if it does occur, then it causes very limited increases in *P. vivax* transmission at the population level.

**Figure 4.**
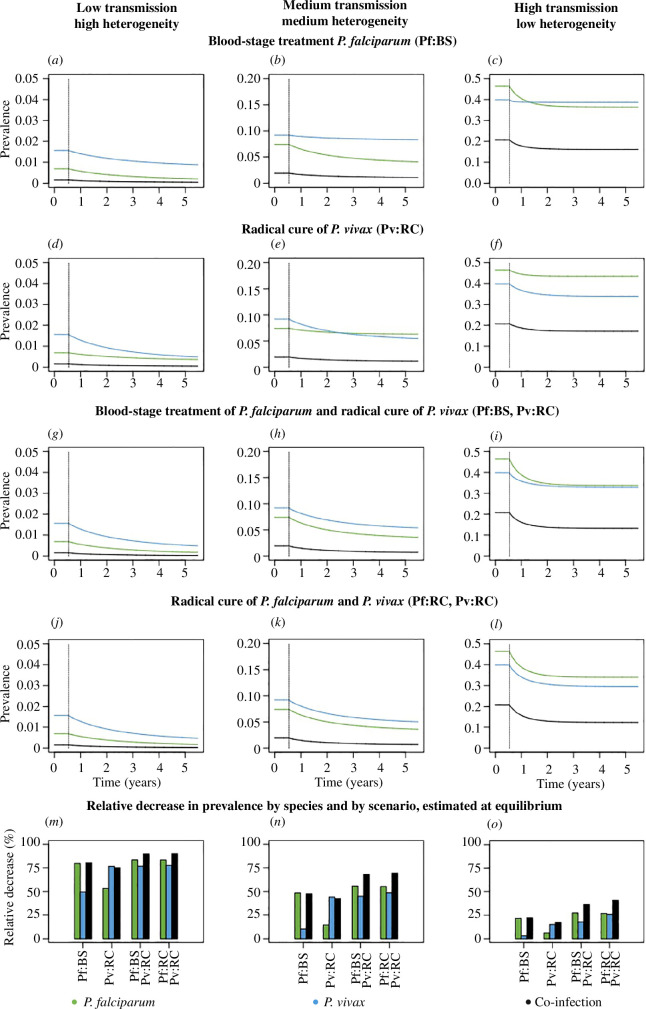
Treatment scenarios. Trajectories of *P. falciparum*, *P. vivax* and co-infection prevalence after the introduction of (*a*–*c*) blood-stage treatment for *P. falciparum*; (*d*–*f*) radical cure for *P. vivax*; (*g*–*i*) blood-stage treatment for *P. falciparum* and radical cure for *P. vivax*; (*j*–*l*) radical cure for both *P. vivax* and *P. falciparum*. For each epidemiological setting and treatment scenario, (*m*–*o*) depict the relative reduction in prevalence of *P. falciparum*, *P. vivax* and co-infections. The left column shows low transmission and high heterogeneity with m=0.27 and $\sigma^{2}=2.4$. The middle column shows medium transmission and heterogeneity with m=0.34 and $\sigma^{2}=1.53$. The right column shows high transmission and low heterogeneity with m=0.63 and $\sigma^{2}=0.6$.

In all settings, *P. falciparum* treatment reduced *P. vivax* prevalence, and *P. vivax* treatment reduced *P. falciparum* prevalence (figure 4*a–f*,*m–o*). Higher levels of heterogeneity were associated with more frequent *P. vivax* and *P. falciparum* co-infection, and a greater effect of treating one parasite on the other. For instance, *P. falciparum* treatment reduced *P. vivax* prevalence by 49.6% versus 2.9% in the high versus low heterogeneity setting, respectively (figure 4*m* versus 4*o*); meanwhile, *P. vivax* treatment reduced *P. falciparum* prevalence by 53.9% versus 6.2% in the high versus low heterogeneity setting (figure 4*m* versus 4*o*).

Treating *P. vivax* with radical cure and *P. falciparum* with blood-stage only treatment leads to a 77.0%, 26.1% and 17.8% relative reduction in *P. vivax* prevalence in the low, medium and high transmission settings, respectively (figure 4*g–i*,*m–o*). Compared with that, treating both *P. vivax* and *P. falciparum* with radical cure leads to a 77.8%, 48.8% and 26.1% relative reduction in *P. vivax* prevalence in the low, medium and high transmission settings, respectively (figure 4*j–l*,*m–o*).

## 4. Discussion

The aim of this study was to develop a compartmental model to investigate co-infections of *P. falciparum* and *P. vivax* while considering heterogeneity in mosquito biting exposure. This model is one of few that captures co-infections and the dependence between *P. falciparum* and *P. vivax* [31,32]. To our knowledge, it is the only one that explores heterogeneity as a driving factor of epidemiological dependence between the species.

To inform model development and fitting, we gathered data from field epidemiology studies documenting the prevalence of *P. vivax*, *P. falciparum* and co-infections through a literature review. In this data, we found a positive linear relationship between *P. vivax* and *P. falciparum* PCR-prevalence. We observed an excess in co-infections, indicating a deviation from expectation under independence, in line with previous findings [40]. This excess in co-infections was more variable at lower transmission intensities, which has previously been observed in the extensive work by McKenzie & Bossert [41,42]. A mechanism that has been proposed to explain excess co-infections is that transmission, especially in low and declining transmission settings, is more heterogeneous [34]. Heterogeneity in transmission probably operates on multiple axes, via biological, environmental and/or socio-behavioural mechanisms. It could be linked to individual differences in host pheromones or CO_2_ emissions, the location of households relative to vector breeding sites, differences in occupational exposure to vector habitats, or in domestic habits, such as the use of bed nets and other protective measures, the herding of animals in proximity of households or domestic water management. Heterogeneity reduces the number of accessible individuals and thus the effective population size, keeping the prevalence over the whole population low, while forcing more co-infections than would be expected for this prevalence. This motivated us to explicitly model heterogeneity in biting exposure. A key limitation was that we did not model heterogeneity as a function of transmission intensity.

We developed a coupled compartmental model with 17 compartments, 16 fixed and 3 estimated parameters, including $\sigma^{2}$, a variance parameter that captures the heterogeneity in biting exposure. We fit the model to the literature review data using ABC. We showed that, while some excess co-infections are expected under a homogeneous model ($\sigma^{2}=0),$ as was shown previously [8,9], a model with heterogeneity ($\sigma^{2}>0$) generated simulated data that better resemble review data. This indicates that heterogeneity is sufficient to capture the dependence between both species. We then conducted scenario simulations to explore two potential mechanisms of additional dependence between *P. falciparum* and *P. vivax*, both of which are of public health relevance.

Because the biological mechanisms of hypnozoite activation and subsequent relapses remain unknown [43], trying to understand them with mathematical modelling is highly relevant [44]. The activation-by-fever hypothesis, as opposed or in addition to spontaneous reactivation, was based on the observation that in therapeutic studies, a clinical episode of *P. falciparum* could significantly predict a *P. vivax* relapse [45–49]. In our simulations, we showed that introducing febrile triggering of *P. vivax* relapses increased *P. vivax* and co-infection prevalence most in the epidemiological setting with high transmission and low heterogeneity. In this setting, assuming all new febrile *P. falciparum* infections activate hypnozoites, resulted in a relative increase of at most 5.3% in *P. vivax* prevalence. While this does not undermine the biological importance of the question, it suggests supplemental activation-by-fever is not significant on a population level.

We showed that treating either *P. vivax* or *P. falciparum* reduced the prevalence of both species and co-infections, particularly in settings with higher heterogeneity. While combining radical cure for *P. vivax* and blood-stage treatment for *P. falciparum* resulted in substantial reductions in prevalence across different transmission settings, additionally administering radical cure to febrile *P. falciparum* would reduce *P. vivax* prevalence even further. This is in line with the results in [31,32]. It should be noted that while the former approach is the treatment guideline, it is currently not implemented in many countries, due to concerns about the dangerous side effects of hypnozoiticidal treatment in glucose-6-phosphate dehydrogenase (G6PD)-deficient individuals and the cost of G6PD screening [50].

It is important to acknowledge the limitations of our work. While the simulations we performed were based on plausible parameters, which were either assumed, derived from literature or estimated using review data, they are meant to predict qualitative rather than quantitative insights. When it comes to the choice of a modelling framework there is always a balance between breadth and precision. By choosing to parsimoniously disentangle the qualitative dynamics of co-infections, we had to opt out of a more complex approach that would explicitly feature age, immunity dynamics, non-pharmaceutical interventions and more.

Regarding the literature review, while studies with zero-prevalence for either species were included, we could not account for a possible publication bias resulting in such studies being underrepresented in the published literature. In our work, this bias would result in an underrepresentation of the points at the origin of the plots in figure 1*b–d*, where observed and expected co-infection prevalence are both 0. However, we see that this space is accessible using our model (figure 2*b*), which indicates that the potential bias has little impact on our main conclusion that a model featuring heterogeneity can capture the full scope of the observed data.

Our model fitting procedure requires certain considerations. First, while it is in line with the state-of-the-art in the malaria modelling literature, we fit equilibrium model values to prevalence data that may not be sampled during steady-state dynamics thereby only capturing average behaviours of *P. vivax* and *P. falciparum* and their co-infections. Then we use ABC, which is normally used to infer the parameters of a model using some moderately large number of identically distributed data points. In our case, we have 58 PCR data points that are not identically distributed: each comes from a unique epidemiological setting. We thus fit the model to each data point in turn, which did not incur additional computational cost under the ABC framework, because it is easily parallelisable. Another valid, though more computationally expensive approach would have been to build a mixed-effects model, where hyperparameters governing the distributions of $\sigma^{2}$, m and φ are jointly estimated. Moreover, ABC can be performed using either a k-nearest neighbor (kNN) or distance approach [51]. Due to the non-uniform distribution of prior sampled points in the three-dimensional state space, we chose a kNN approach rather than a fixed distance measure for all 58 parallel iterations of the ABC algorithm. While results under the two approaches are comparable, we acknowledge that with the kNN approach, the posterior precision is not equally interpretable for all 58 points in the posterior ensemble. Finally, as we are estimating a three-dimensional parameter space based on a range of three-dimensional data points, we acknowledge that there is an issue of identifiability, especially at very low prevalences, and where the data are less informative. However, as we are using our parameter estimates for a qualitative interpretation of simulated prevalence trends, this does not greatly influence our main conclusions.

Our findings have the potential to inform the development of complex agent-based models of co-infections. We have shown that a very simple model featuring a few fundamental dynamics can aptly capture the epidemiological dependence between *P. vivax* and *P. falciparum*, if it explicitly models heterogeneity in biting exposure. The modelling framework we present and the insights gained on co-infection epidemiology through the literature review can serve as a novel community resource for exploring public health approaches in coendemic regions of the world. Predictions from more complex models based on our framework could inform case management strategies for both *P. vivax* and *P. falciparum*, including *Pv*SeroTAT, a novel serological testing and treatment strategy [52].

## Data Availability

The literature review data and an excel sheet describing all studies included after title and abstract screening, and all R-scripts, are available [53]. Supplementary material is available online [54].
